# Age‐related changes in the fiber structure around adipocytes in the subcutaneous fat layer and their association with skin viscoelasticity

**DOI:** 10.1111/srt.13566

**Published:** 2024-01-25

**Authors:** Koji Mizukoshi, Motonori Kurosumi, Yoshihiro Hamanaka

**Affiliations:** ^1^ POLA Chemical Industries, Inc. Yokohama Kanagawa Japan

**Keywords:** adipocyte, fiber, scanning electron microscope, subcutaneous fat layer, viscoelasticity

## Abstract

**Objective:**

Age‐related changes in the fiber structure around adipocytes were investigated via scanning electron microscopy (SEM) of excised skin tissues. In addition, the viscoelasticity of the subcutaneous fat layer was evaluated via elastography, and the association between the fiber structure and the viscoelastic properties was assessed.

**Methods:**

Skin tissues excised from the facial cheek area were used. Then, SEM images of these tissues were obtained. The thickness and quantity of the fibers around adipocytes were assessed using a 5‐point scale. The score was used to grade 18 tissue samples. Moreover, the viscoelasticity of the subcutaneous fat layer in the same samples was evaluated via ultrasound elastography.

**Results:**

Based on the SEM image score, an association was observed between the fiber status score and age, thereby indicating a tendency toward age‐related fibrosis. Fiber structures with high scores, which indicate fibrosis, had a significantly lower viscoelasticity based on ultrasound elastography.

**Conclusion:**

The thickness and quantity of fibrous structures around adipocytes in the subcutaneous fat layer increase with age, and these changes can be associated with decreased viscoelasticity in the subcutaneous fat layer.

## 1 INTRODUCTION

The softness and elasticity of the skin decrease with age, and improvement in these sensations is a major cosmetic target.^1^ The skin has a multilayered structure. Humans perceive softness from sensation based on the extent of pushing and bouncing when a finger touches the skin surface. Thus, the physical viscoelastic properties of each of the multilayer structures are important for the sensation of softness. Previous studies have shown that the viscoelasticity of the stratum corneum/epidermis, dermis,^2^ and subcutaneous tissue,^3^ which comprise the skin layer, decreases with age. However, most studies have focused on changes in the viscoelasticity of the stratum corneum/epidermal and dermal layer. Keratin and moisturizing substances have effects on the stratum corneum/epidermis.^2^ Some studies have shown quantitative and qualitative age‐related changes in fibrous tissues in the dermal layer.^4^ Others have shown that such changes are caused by ultraviolet radiation. Moreover, changes in the fibrous structure are associated with viscoelasticity.^5^

The condition of the subcutaneous fat layer, which is approximately five times thicker than the dermis,^3^ has a significant effect on tactile sensation. Therefore, histological factors associated with age‐related viscoelastic changes in the subcutaneous fat layer should be elucidated. However, histological aging‐related changes in the subcutaneous fat layer have not been validated in detail until recently. Recently, it was shown that the structure of the border region between the dermis and the subcutaneous fat layer changes with age.^6^ The longitudinal retinacula cutis (RC) structure of the subcutaneous tissue changes with age, and it is associated with skin viscoelasticity.^7^ Scanning electron microscopy (SEM)^8^ and tissue staining^9^ of the subcutaneous fat layer have revealed the presence of a mesh‐like fibrous structure around adipocytes, which is collagen, in addition to the structures described in previous studies. However, age‐related changes in this fiber structure and their effect on viscoelasticity have not been evaluated.

This study examined individual differences and age‐related changes in the fibrous structure around adipocytes via SEM of skin tissues excised from human cheeks. Moreover, local viscoelastic properties were assessed via ultrasound elastography using the same tissue samples, and the association between the viscoelastic properties and the fibrous structure around adipose tissues was analyzed.

## 2 MATERIALS AND METHODS

### 2.1 Human skin tissue acquisition

**FIGURE 1:**
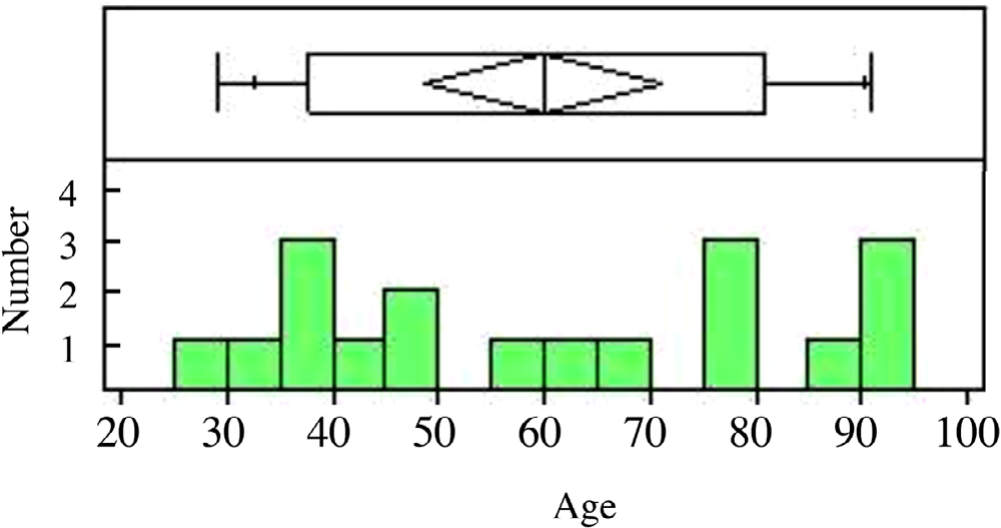
Histogram of the age distribution of skin tissue donors used in the study.

Human cheek skin tissues, including epidermis, dermis, and subcutaneous adipose tissues, were purchased from Obio, LLC (El Segundo, CA, USA). The tissues were collected from 18 Caucasian adult women aged 20−100 years. Then, they were frozen. Prior to obtaining human tissue samples, we ensured that Obio, LLC complied with all ethical and applicable laws, rules, and regulations. Moreover, the company obtained a written informed consent from each donor. Figure 1 shows the number of samples and ages.

### 2.2 Preparation and assessment of subcutaneous tissues via SEM

The samples were prepared based on methods used in a previous study^8^ that performed microstructural analysis with SEM. The following is a brief description of the procedures: Using a microtome blade, the samples were shredded while frozen on dry ice. Next, they were immersed in phosphate buffered saline (PBS) (−) for thawing. The tissue was cracked with tweezers to create a section with the internal structure exposed to retain the adipocyte structure. Under this condition, the tissues were immersed in 2% glutaraldehyde ‐ 2% paraformaldehyde and treated overnight at 4°C. After washing with PBS (−), the tissues were immersed in 1% osmic acid solution and treated for 3 h at 4°C. Then, they were washed again with PBS (−) and dehydrated with 50%−100% ethanol. The tissue was treated with a mixture of ethanol and tert‐butyl alcohol and was vacuum freeze‐dried. Next, the tissues underwent platinum deposition using an ion sputter. To assess the fibrous structure around adipocytes in the fat layer at magnification of 500×, SEM images were acquired using JSM‐IT500HR (JEOL, Tokyo, Japan). SEM image acquisition of each donor tissue was performed from the upper and lower subcutaneous fat layer, each with three images, with a total of six images.

### 2.3 Evaluation of SEM images

Using all SEM images, the order was established by focusing on the quantity and thickness of the fibers around adipocytes. The sequential order was then divided into five segments, and the representative images were selected from each of the five segments. These images were used to produce a fiber state score around adipocytes in the subcutaneous fat layer at five levels. The scores of each donor sample were then evaluated by the following method using scores generated. Three reviewers scored all six SEM images obtained per donor. The average value of each of the six images was calculated and used as the score of each donor. All reviewers performed the evaluation under the same conditions with the SEM images displayed at equal magnification on the same display.

### 2.4 Viscoelasticity assessment via elastography

Tissue viscoelasticity was evaluated using donor tissues utilized in SEM studies with the HITACHI Noblus ultrasound elastography system (Hitachi, Japan). Ultrasound probes of 8−15 M Hz were used. The size of the probe surface was generally 1 × 5 mm. Only the donor tissue with a sufficient skin surface area relative to the size of the measurement plane of the ultrasound probe was used. Eight samples satisfied the condition. Of these samples, four with the highest fiber status scores ratings made in the previous section were included in the high score group and four samples with the lowest fiber status scores in the low score group. The mean viscoelasticity values of the high and low score groups were compared. The thickness of skin samples was evaluated with a ruler under a frozen state, and the underside of the tissue was trimmed to ensure that the thickness of all samples was similar. Elastography measurements were based on methods in a previous study.^3^ While keeping the ultrasonic probe in contact with the skin surface, a rectangular reciprocating motion was performed using a motorized slider (LES8DK‐50‐R36ND, SMC, Japan) with a push‐in distance of 0.3 mm, a push‐back speed of 30 mm/s, and an interval of 1 s. In the current analysis, data during push‐in were used for analysis. A standard coupler (Hitachi, EZU‐TECPL1) was set between the probe and the skin. All measurements were divided by the standard.

### 2.5 Analysis of ultrasound elastography data

**FIGURE 2:**
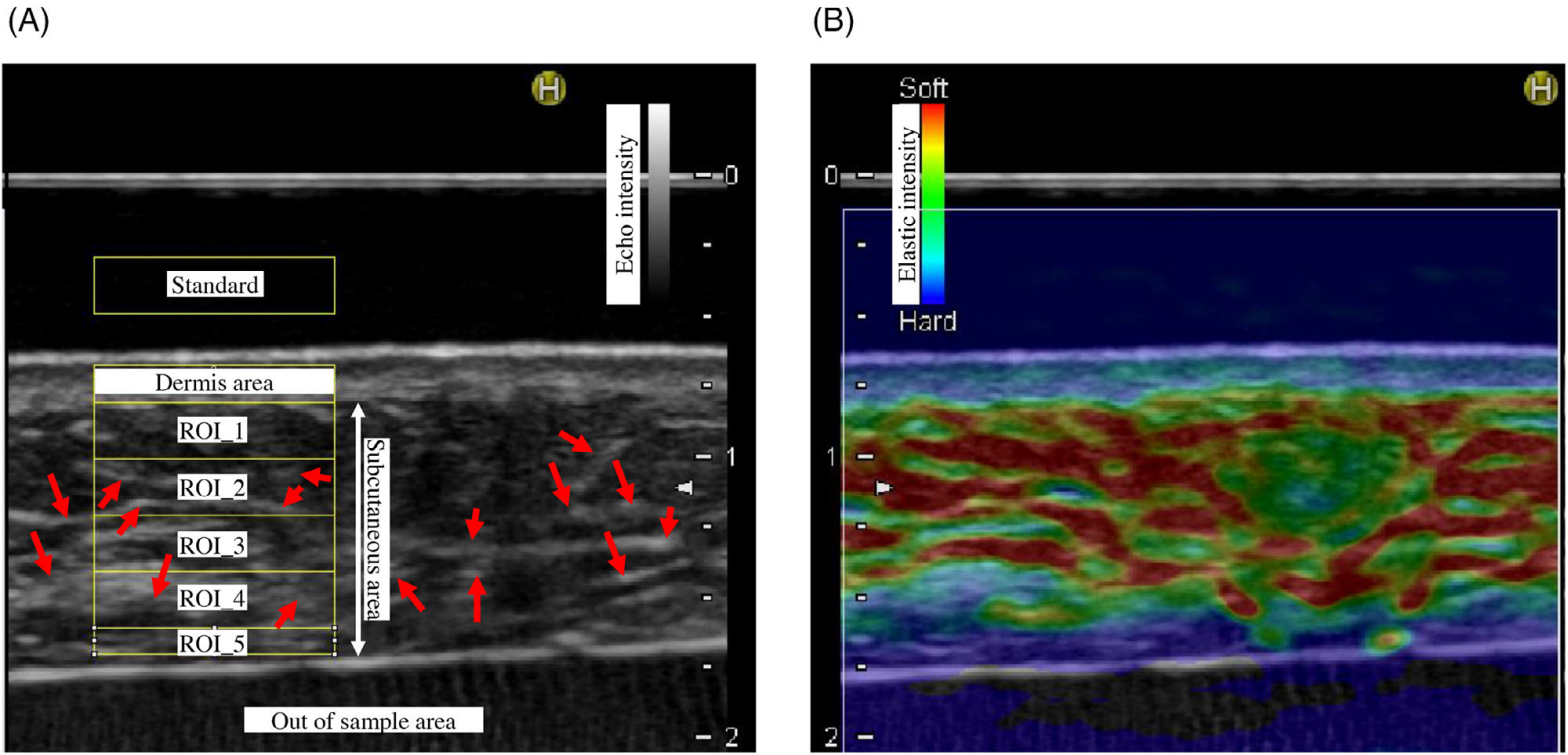
Typical examples of ultrasound and ultrasound elastography images. (A) Setting the analysis area performed on an ultrasound image. A region of interest (ROI) with a thickness of 0.23 mm and a constant width was set to be tangential to the border region between the dermis and subcutaneous fat layer (ROI_1). The ROI was set to the lower edge of the tissue using the same method. The red arrows indicated fiber structures across the subcutaneous tissue. (B) Ultrasound elastography image of the same area as in (A).

The analysis area of the ultrasound elastography image was set in the direction of the depth of the skin cross‐section. Figure 2 depicts the representative example of ultrasound measurement and ultrasound elastography images obtained using Noblus. The analysis area was set using the ultrasound images of each participant (Figure 2A). A constant width region of interest (ROI) with a depth of 0.23 mm was set up tangential to the border region between the dermis and subcutaneous fat (ROI_1). The dermal and subcutaneous fat layer boundaries were established according to the methods used in previous studies.^3^ The ROI was set to the lower edge of the tissue image using the same method. In the subcutaneous fat layer, there were fibrous structures (indicated by arrows in Figure 2A) at a structural level greater than the pericellular meshwork that traverse extensively throughout the layer. Therefore, to analyze the association between the viscoelastic properties and the reticular fibrous structure around adipocytes, this study used ROI_1 measurements in the upper subcutaneous fat layer, where such a large level of fibrous structure is rarely observed. Each ROI was superimposed on the ultrasound elastography image (Figure 2A), and its elastography value was calculated. The results were derived only on the image with 256 gradations. Therefore, in the measurement region, the average measurement value of the ultrasound elastography represented by 256 gradations was calculated. These analyses were performed using ImageJ (National Institutes of Health, Bethesda, Maryland, USA). All values were divided using the standard calculated value to compare the measured values between donner samples. Based on the abovementioned analysis method, the ultrasonic elastography measurement (a.u.) value was between 0 (low viscoelasticity) and 255 (high viscoelasticity).

### 2.6 Statistical analysis

All statistical data were analyzed using the JMP software version 19 (SAS Institute, Cary, NC, USA). A correlation analysis between measurement values was performed using spearman rank correlation coefficient. The unpaired *t*‐test was used to evaluate differences between measurement values.

## 3 RESULTS

**FIGURE 3:**
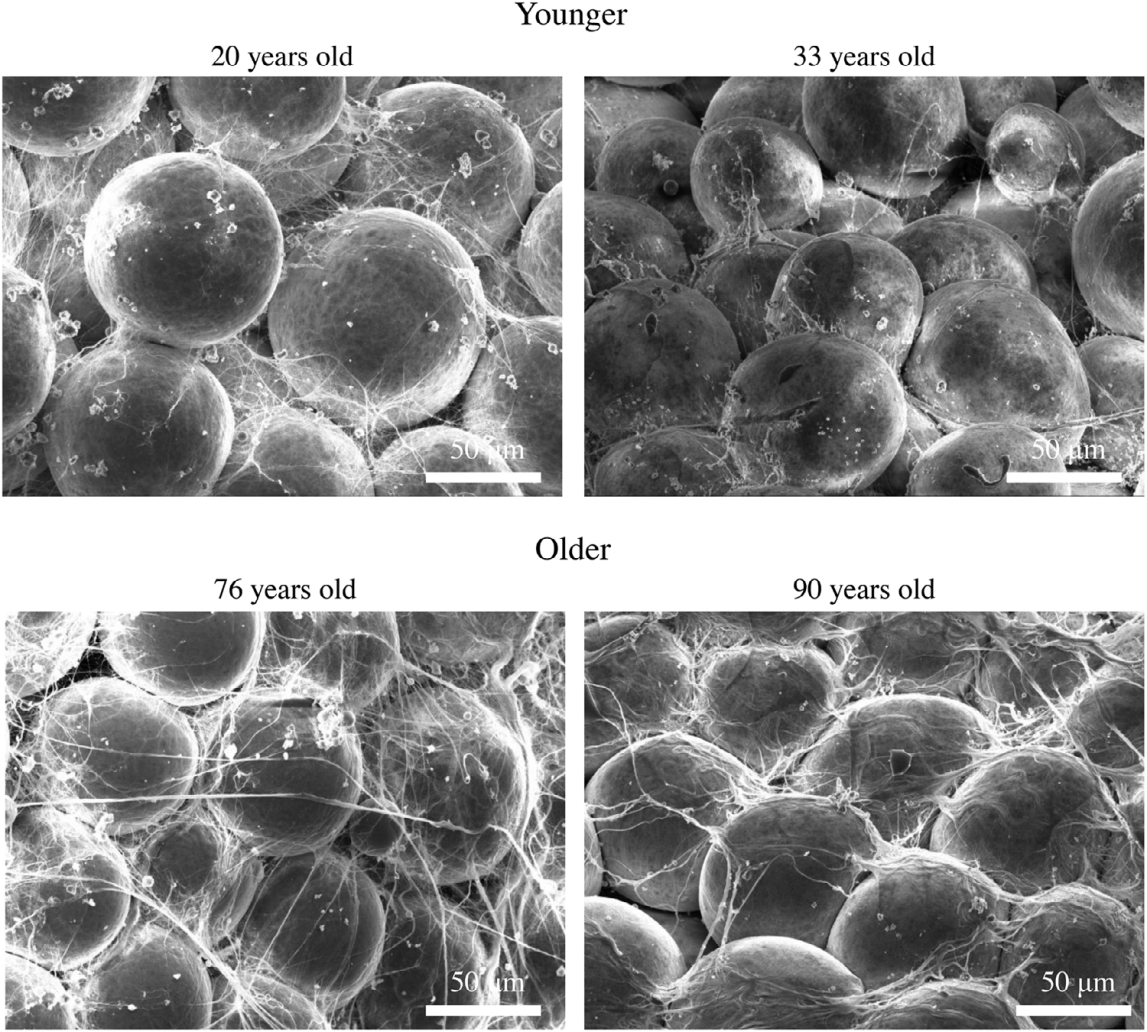
Representative scanning electron microscopy (SEM) image of the fiber structure around adipocytes in the subcutaneous fat layer. (A) Representative SEM image of the younger age group. (B) Representative SEM image of the older age group.

Figure 3 shows the representative SEM images of young (20−33 years old) and old (76−90 years old) subcutaneous fat layer. In almost all images, the spherical adipocytes were surrounded by a network of fibers. However, the thickness, which indicates fiber quality, and the quantity, which represent fiber abundance, varied per sample. Comparing the young and old samples showed that younger samples had finer fibers in the intercellular spaces, whereas older samples had thicker fibers covering more of the cell surface both quantitatively and were more abundant outside the intercellular spaces.

**FIGURE 4:**
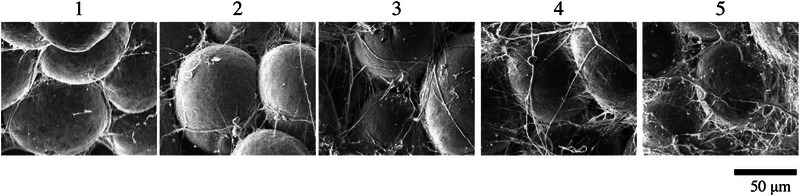
Image of the five‐scale score of the fiber structure around adipocytes. If the score was 1, the fibers around adipocytes were thin and found only between cells. As the score increases, the fiber structures were thicker and more extensive in the field of view.

Hence, based on the abovementioned results, the characteristics of the fibrous structure around adipocytes changed with age. Therefore, the characteristics of fibers were scored for a more quantitative evaluation. The field of view of the score image was cropped to a region containing a few cells to represent the viewpoint of the fibrous structure around adipocytes more accurately. For cropping, the area with the most average features in one SEM image was selected, and all images were cropped to the same size. In total, 108 SEM images, with six from one donor, were ordered based on the overall perspective of the quantity and thickness of the fiber structures. The whole sequence was divided into five stages. Then, a representative image was selected from each stage and set as the five‐stage fiber status score, as shown in Figure 4. If the score was higher, the fibers had a higher quantity and were thicker.

**FIGURE 5:**
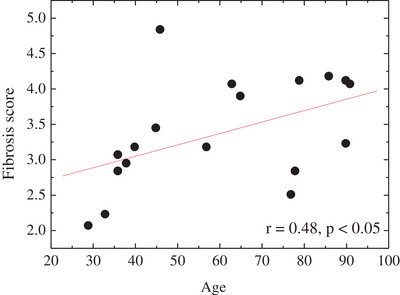
Age‐related changes in the fiber structure between adipocytes. The scores were more likely to increase with age.

The scores were used to evaluate the SEM images of 18 donors. Score evaluations were performed by setting up a field of view area equal to the score image in the area with the most average features in the SEM image that should be evaluated, as shown in Figure 5. A correlation was observed between fiber status score and age (*r* = 0.48, *p* < 0.05).

**FIGURE 6:**
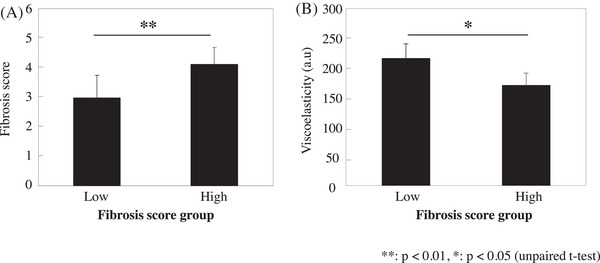
Association between fiber structure status based scanning electron microscopy and viscoelasticity measured via elastography. (A) Significant differences in fiber status scores between the low and high score groups. (B) Significant differences in viscoelasticity in the subcutaneous fat layer between the low and high score groups.

Of eight samples available for elastography, four with high fiber status scores were included in the high score group and four samples with low fiber status scores in the low score group. Figure 6A depicts the mean fiber status scores of the high and low score groups. The fiber status scores of the two groups significantly differed. Figure 6B shows the mean viscoelasticity values on elastography between the two groups. The high score group of fiber status had a significantly lower viscoelasticity than the low score group.

## 4 DISCUSSION

Using skin tissues excised from human cheeks, we examined age‐related changes in the fibrous structure around adipocytes in the subcutaneous fat layer. Moreover, the association between the fibrous structure and the viscoelastic properties was evaluated.

Based on the SEM of the fiber structures around adipocytes, the quantity and thickness of fiber structures might change with age (Figure 3). Therefore, to establish an index to more quantitatively evaluate the extent of this change, a scoring system for the quantity and thickness of the fiber structure was established (Figure 4). The order was performed by synthesizing the viewpoints of the quantity and thickness of the fibers. Result showed that the fiber status score varied proportionally in terms of quantity, from less to more fibers, and thickness, from thinner to thicker fibers. These changes could reflect fibrosis. Using the generated scores, the SEM images of 18 participants were evaluated, and the scores were more likely to change with age (Figure 5). Thus, fibrosis, defined as an increase in the thick fiber, might have developed. In the subcutaneous fat layer, in addition to the fiber structure around adipocytes, which is the focus of this study, there was also an RC structure that traverses a wide area of the whole layer, and this structure decreases with age.^7^ The current study showed that fiber structures at different size levels in the subcutaneous fat layer might change with age. Further, we examined the facial skin, which is considered important cosmetically. Nevertheless, future studies should assess whether these age‐related changes are systemic. In the skin, particularly in the dermal layer, UV light affects the fiber structure.^5^ However, it only reaches the dermal layer.^10^ Therefore, the direct effects of ultraviolet rays are minimal. Cellulite is a fiber structural change in the tissue of the subcutaneous fat layer. Pathological findings include enlarged adipocytes and increased collagen fibers that bind to multiple adipocytes. In addition, the vascular structures are compressed.^11^ Thus, fibrosis in the subcutaneous fat layer may be associated with oxygen saturation. In fact, another study conducted by the authors has shown that oxygen saturation in the skin decreases with age.^12^ In the future, a more detailed investigation about the causes of fibrosis in the subcutaneous fat layer of the face should be conducted using different techniques such as in vitro cell experiments under hypoxic conditions.

Then, to examine the association between changes the fibrous state around adipocytes in the subcutaneous fat layer and the viscoelastic changes in the tissues, ultrasound elastography was performed to examine the viscoelastic tissue fragments. The presence of fibrous structures in the subcutaneous fat layer at a size reflected in the echo intensity was confirmed on ultrasonography. The fibrous structures were found over a wide area in the subcutaneous fat layer (as indicated by red arrows in Figure 2A). The fibrous structures observed in a wide range of layers in the ultrasonography image were widespread fibrous structures such as the RC. The current study examined the effect of the viscoelasticity of finer fibrous structures around adipocytes that might not be detected on ultrasonography. Therefore, ROI_1, which is the uppermost layer of the subcutaneous fat layer, where this extensive fiber structure is not observed, was used in the current study. The viscoelasticity of ROI_1 was evaluated via elastography. Results showed that viscoelasticity was more likely to decrease in the high score group, which had more thick fiber structures (Figure 6). The fiber structure around adipocytes could affect viscoelasticity. Our previous studies have shown that the viscoelasticity of the upper subcutaneous fat layer decreases with age.^3^ Further, age‐related decrease in the viscoelasticity of the upper subcutaneous fat layer may be affected by changes in the fiber structure around adipocytes. In the current study, the number of samples with a sufficient area for elastography was limited by the use of excised skin tissues, and it was not possible to obtain age correlations when evaluating the association between fiber structure scores and viscoelasticity. Therefore, a more detailed study with a larger sample size should be conducted.

The current study showed that not only the dermis but also the subcutaneous tissue were important for maintaining skin softness and elasticity. However, these properties decrease with age,^1^ and their improvement is a major cosmetic target. The condition of the subcutaneous fat tissue in the cheeks, which is about five times thicker than the dermis, can significantly affect skin softness. Nonetheless, future studies should assess the mechanism associated with changes in fiber structures.

## 5 CONCLUSION

Based on the SEM of excised skin tissues, the thickness and quantity of the fiber structures around adipocytes in the subcutaneous fat layer were more likely to increase with age. This finding indicated the possible development of fibrosis. Moreover, ultrasound elastography revealed that changes in the fiber structure might be associated with decreased viscoelasticity in the subcutaneous fat layer.

## CONFLICT OF INTEREST STATEMENT

The authors declare that there is no conflict of interest that could be perceived as prejudicing the impartiality of the research reported.

## Data Availability

The data that support the findings of this study are available on request from the corresponding author. The data are not publicly available due to privacy or ethical restrictions.
